# Medical management of cerebral edema in large hemispheric infarcts

**DOI:** 10.3389/fneur.2022.857640

**Published:** 2022-11-04

**Authors:** Grace DeHoff, Winnie Lau

**Affiliations:** ^1^Department of Neurology, University of North Carolina, Chapel Hill, NC, United States; ^2^Department of Neurosurgery, University of North Carolina, Chapel Hill, NC, United States

**Keywords:** hemispheric stroke, cerebral edema, hyperosmolar, herniation, glibenclamide

## Abstract

Acute ischemic stroke confers a high burden of morbidity and mortality globally. Occlusion of large vessels of the anterior circulation, namely the intracranial carotid artery and middle cerebral artery, can result in large hemispheric stroke in ~8% of these patients. Edema from stroke can result in a cascade effect leading to local compression of capillary perfusion, increased stroke burden, elevated intracranial pressure, herniation and death. Mortality from large hemispheric stroke is generally high and surgical intervention may reduce mortality and improve good outcomes in select patients. For those patients who are not eligible candidates for surgical decompression either due timing, medical co-morbidities, or patient and family preferences, the mainstay of medical management for cerebral edema is hyperosmolar therapy. Other neuroprotectants for cerebral edema such as glibenclamide are under investigation. This review will discuss current guidelines and evidence for medical management of cerebral edema in large hemispheric stroke as well as discuss important neuromonitoring and critical care management targeted at reducing morbidity and mortality for these patients.

## Introduction

Acute stroke effects ~795,000 persons in the United States annually of which 87% are acute ischemic strokes (1). Large hemispheric infarcts (LHI) are defined variably in the literature but are typically thought of as infarct that involve one-half to two-thirds of the anterior circulation territory (2–5) and comprise 7.6% of acute ischemic strokes (6). Large vessel occlusions make up 26–38% of ischemic strokes and of these patients 22–50% will result in LHI with significant cerebral edema (7). The danger of LHI is the formation of malignant cerebral edema (MCE), which in a fixed cranial vault, poses a risk of further compression of normal brain tissue and herniation. Risk factors for development of MCE include young age, higher NIHSS and size of hypoattenuation on admission CT (8). The large majority of these patients will progress to neurologic deterioration within 72 h of symptom onset and mortality without surgical intervention is high at ~80% (9, 10).

In this paper we reviewed the literature from Pubmed, Google Scholar, and Medline as well as Clinicaltrials.gov utilizing a search strategy including the keywords large hemispheric stroke, malignant stroke, malignant cerebral edema, large core infarct, and hyperosmolar therapy to identify pertinent literature. This paper will discuss the physiology and formation of edema in LHI and the evidence behind various treatment strategies including hyperosmolar therapies and other pharmacologic therapies. The evidence and utility of surgical interventions for LHI are discussed elsewhere in this issue of Frontiers in Neurology. Prognosis and survival of LHI are also heavily influenced by critical care management aimed at minimizing complications such as infection and venous thromboembolism. Advance care planning and best communication practices in shared decision-making with patients and families also significantly affect outcomes (11).

## Pathophysiology

Cerebral edema is the pathological formation of excess fluid in tissue surrounding an injured region of brain. In the fixed cranial vault, edema contributes to herniation risk as it compresses healthy brain tissue into the cistern spaces. As the pressure in the tissue increases, it eventually will exceed the capillary pressure further reducing capillary inflow and cerebral perfusion (12, 13). The type of edema that forms is dependent on the type of neurological insult and often involves multiple processes: ionic, vasogenic, cytotoxic and hydrostatic.

At the cellular level, cytotoxic edema is the first process to occur and sets up the gradients for development of ionic and vasogenic edema. In healthy tissue, sodium resides in the extracellular space and is a key ion in voltage-dependent channels of neurons and astrocytes. Ischemia causes a dysfunctional energy state with dysregulation of sodium transport channels and results in loss of normal biochemical gradients. The intracellular accumulation of sodium then triggers an upregulation of sodium channels (12). The sulfonylurea receptor 1 (SUR1) channel is a non-selective cation channel and is upregulated within 2–3 h of ischemia and is a target of therapy for MCE (14). The opening of this channel results in a net flow of sodium to the intracellular space (15). As the intracellular sodium concentration rises, osmotic forces pull water into the cell *via* Aquaporin-4 (AQP4) channels (16). This cytotoxic edema will lead to cellular lysis and necrotic cell death and a resultant increase in extracellular sodium (12). Early ionic edema is driven by the movement of water into the sodium rich extracellular space from the intravascular space, however the blood brain barrier (BBB) remains intact against the movement of macromolecules during the early ischemic phase predominated by ionic edema (12).

Disruption of the BBB is the hallmark of vasogenic edema; however the full mechanism of injury is a point of active research. Initial breakdown occurs between neighboring endothelial cells when proteins like thrombin trigger endothelial cell retraction. Up-regulation of vascular endothelial growth factor (VEGF) in isolated perfused micro vessels contributes to the uncoupling of tight endothelial junctions and results in increases hydraulic conductivity (17). Matrix metalloproteinases (MMP) are also upregulated by ischemia and contribute to the degradation of the microvascular matrix of the basement membrane, but the full pathway is unknown (18). MMP inhibitors have been shown to reduce ischemia and edema *in vivo* in rats (19). MMP has also been studied as a measurable biomarker for cerebral edema (12). These pathways promote communication between the intravascular and cerebral interstitial space allowing water and plasma proteins to extravagate into the cerebral interstitial space (13, 20). Several molecular biomarkers including NEU, TNF-alpha, IL-1b, IL-6, IL-8, IL-17, ICAM-1, VCAM-1, MPO, NE, MMP-9, miRNA, free dsDNA, H3CIT and NETosis are under investigation for their predictive role in MCE after LHI (Clinicaltrials.gov NCT03703284). Recent studies have looked at the contribution of glymphatic flow in the formation of cerebral edema. Mestre et al. studied *in vivo* rat models and human autopsies and found that CSF flow through the perivascular spaces increased within minutes of ischemia and coincided with the onset of swelling and brain water content (21).

## Neuromonitoring

The cornerstone of neuromonitoring for cerebral edema after LHI is the neurologic exam and neuroimaging. Many studies have attempted to combine clinical and radiographic factors to develop clinical prediction scores for the risk of MCE, including the DASH, EDEMA, and E-Score (22–25). In patients with revascularization, pre-procedure factors associated with a higher incidence of MCE include an ASPECTS score of <8, prolonged time to reperfusion, incomplete recanalization, proximal internal carotid occlusion, and poor collateral vasculature (7, 26). Admission computed tomography (CT) is available for the majority of patients presenting with acute stroke symptoms. Quantitative infarct volume measurements by net water uptake (NWU) on admission CT can help identify patients who will progress to LHI and MCE (27). Quantitative measurements CSF volume change (ΔCSF) from baseline to 24 h CT can be an early indicator for risk of MCE, with 10% change almost doubling the risk (28, 29). Combining high risk clinical characteristics with measurements of CSF reserve, and ΔCSF perform well in predictive modeling with AUROC 0.96 (30). The Automatic PredICtion of Edema After Stroke (APICES) trial is currently enrolling patients to evaluate the combination of these features, as well as collateral status, clot burden scores, and vein scores too develop a predictive model for those at risk of developing MCE (NCT04057690). CT perfusion is increasing in use since the recommended extended thrombectomy window for anterior circulation strokes (31). Blood brain barrier permeability maps calculated from initial perfusion imaging may play a role in predicting those who will progress to MCE (32).

Invasive intracranial monitoring, though appealing, has limited utility in LHI. Studies have demonstrated that invasive intracranial monitoring for elevated ICP in hemispheric stroke is limited due to separation of the intracranial compartments, and often herniation occurs prior to elevated ICP. Therefore, routine invasive intracranial monitoring is not recommended (20, 33, 34). Transcranial doppler is a non-invasive option to detect progression of cerebral edema by following trends in the pulsatility index and also allows for calculation of CPP (27, 35). Quantitative EEG is also an increasingly available resource, studies have demonstrated early changes in asymmetrical EEG suppression to potentially precede herniation by hours (36).

## Hyperosmolar therapies

A common treatment strategy for elevated intracranial pressure (ICP) due to cerebral edema is hyperosmolar agents including hypertonic saline and mannitol. It is a common misconception that hypertonic therapies work at the site of edema. Hyperosmolar therapies require an intact BBB to exert their osmotic effects, therefore are not active at the site of edema. The changes in cerebral volume occur instead in healthy brain tissue which subsequently results in lowering ICP (37).

Hyperosmolar therapies act by moving water across an osmotic gradient between the cerebral vasculature and cerebral interstitial space. The efficiency of a hyperosmolar agent is graded by its reflection coefficient. As some agents can permeate through the BBB into the brain parenchyma, a reflection coefficient indicates the selectivity of the intact BBB for these substances. A grade of 0 indicates complete permeability and a grade of 1 indicates complete exclusion of movement across the BBB. Hypertonic saline and mannitol have a reflection coefficient of 1 and 0.9, respectively (38). There is a second proposed physiologic effect that hyperosmolar therapy reduces cerebral blood flow by augmentation of cerebral perfusion pressure in healthy areas of brain tissue with intact autoregulatory vasculature (39, 40). Diringer et al. evaluated cerebral blood flow studies by PET which do not demonstrate a reduction in blood flow, supporting the more dominant effect of decrease brain water content as the ICP lowering mechanism (41).

Various strategies are described for the use of hypertonic saline including bolus therapy vs. continuous infusion, or a combination. Available concentrations are institution dependent. Properties of the most common concentrations 3 and 23.4% are outlined in Table 1. Mannitol 20% solution is administered as weight-based dosing of 0.25–1g/kg, with repeat dosing of 0.25–0.5g/kg while monitoring the osmolar gap. An osmolar gap of >20 mOsm/kg may suggest inadequate renal clearance and should prompt discontinuation of mannitol (42). There are limited prospective trials evaluating hypertonic saline as compared to mannitol in acute ischemic stroke however some studies may suggest more rapid or sustained ICP lowering with hypertonic saline, but neither has demonstrated improvements in functional outcome or mortality in stroke (42). In traumatic brain injury, there is data to support superior ICP lowering effect of hypertonic saline without any differences in neurologic outcomes or mortality compared to mannitol (43, 44). Consideration of individual medical comorbidities, contraindications, and institutional comfort typically guide the decision of which agent is utilized. Hypertonic saline adverse effects include transient hypotension followed by increases in cardiac output, hyperchloremia, and pulmonary edema (45). Some institutions may require administration of higher concentrations of hypertonic saline *via* central access or intraosseous access (46, 47). Mannitol adverse effects include accumulation in renal failure, hypotension and diuresis (48). Prophylactic sodium targets and prophylactic mannitol therapy in absence of signs or symptoms of elevated ICP have insufficient data to support preventing ICP crisis, and may have risks of rebound intracranial hypertension in some studies (20, 34, 49).

**Table 1:** Properties of hyperosmolar agents for treatment of elevated intracranial pressure.

**% Solution**	**Osmolarity (mOsm/L)**	**Sodium concentration** **(mEq/L)**	**Bolus volume for elevated ICP**	**Administration access**
0.9% NaCl	308	154	n/a	Peripheral
3% NaCl	1,026	513	250–500 ml	Peripheral, central, intraosseus
23.4% NaCl	8,008	4,004	15–30 ml	Central, intraosseus
20% Mannitol	1,100	n/a	0.25–1 g/kg	Peripheral

## Glibenclamide and other investigational therapies

The SUR1-TRPM4 protein receptor has recently emerged as a target for prevention of MCE. The receptor, which is upregulated in various forms of central nervous system injury including ischemia, is implicated in the formation of astrocyte edema due to depolarization of the cell and influx of Na+ and subsequent water influx *via* Aquaporin-4 channels (13, 50, 51). Intravenous glibencamide binds to the SUR1-TRPM_4_ protein to impair this process and acts to inhibit the secretion of MMP-9 on endothelial cells and reduce plasma MMP-9 levels in patients with LHI (12, 13).

The GAMES-RP trial compared glibenclamide to placebo in subjects with severe anterior circulation ischemic stroke (52). The trial was negative with regards to the primary endpoint of 90 day mRS of 0–4 without decompressive craniectomy between the glibenclamide and placebo groups (39 vs. 41%, respectively; adjusted *P* = 0.77). Similarly, regardless of progression to surgical intervention, there were no statistical differences in early mortality at 7 days and 90 days, but significantly reduced mortality at 30 days (*P* = 0.03). In a *post-hoc* analysis of patients ≤ 70 years old, there was a statistically significant decrease in mortality at all time points (12, 13). Glibenclamide compared to placebo was associated with a significant decrease in radiographic midline shift and total MMP-9 (52, 53). Vorasayan et al. evaluated the lesional NWU on CT in patients treated with glibenclamide, and demonstrated reduced NWU (*P* = 0.016) in both gray and white matter, and reduced midline shift (*P* = 0.016) (54). Despite these promising radiographic and biomarker findings, glibenclamide did not reduce the use of hyperosmolar therapy in the trial (55). The phase three CHARM Trial is a randomized, double-blind, placebo-controlled, parallel-group, multicenter study is currently underway (Clinicaltrials.gov NCT02864953).

Downstream targets such as aquaporin-4 channels are also being investigated as therapeutic modulation of cerebral edema. Arginine vasopressin is thought to play a role in the upregulation of these channels *via* the V1 receptor (56, 57). Vasopressin antagonism with vaptan is currently being studied in cerebral edema secondary to intracerebral hemorrhage but requires further investigation (Clinicaltrials.gov NCT03000283).

## Surgical management

Decompressive hemicraniectomy (DCHC) is the surgical removal of the skull over the affected hemisphere to allow for expansion of cerebral edema extracranially and lessen the herniation risk. The full discussion regarding surgical management of large strokes is covered elsewhere in this issue of Frontiers in Neurology and only covered briefly in this article. In a select patient population age 60 and under, DCHC is associated with lower mortality (adjusted odds ratio, 0.16%; 95% CI, 0.10–0.24) and increased chance of favorable outcome (adjusted odds ratio, 2.95; 95% CI, 1.55–5.60). Favorable outcome was defined as mRS score ≤ 3 at 6 months and 1 year with a shift toward functional improvement (10).

## Other critical care management

Common practices to reduce ICP in neurocritically ill patients include physical maneuvers including elevation of the head and hyperventilation. The mechanism for these interventions decreases ICP through mechanical and structural changes but does not have any impact on the volume of cerebral edema or swelling. For acute elevations in ICP concerning for imminent herniation, transient hyperventilation to a goal PaCO2 of 25 mmHg can be temporizing until definitive intervention (20, 58). This intervention is time limited as prolonged cerebral vasoconstriction has been associated with ischemia. Patients being treated for cerebral edema should have the head of bed elevated to 30 degrees, but no more than 45 degrees as an adjunct method for reduction of ICP (20). Clinicians should be cognizant of prolonged positioning in trendelenburg with the head of bed lower than the heart or rotated as this can result in increases in ICP from compression of cerebral venous outflow (59). Other therapies that have been studied but demonstrated to be non-beneficial for management of cerebral edema in LHI include corticosteroids (60) and therapeutic induced hypothermia (20, 33, 34).

Patients with LHI are at high risk for compromise of their airway and at an increased risk for pulmonary complications such as aspiration pneumonia (61). Stroke associated pneumonia (SAP) can be a contributor to morbidity and potential mortality in patients with large hemispheric strokes. Early recognition and empiric antibiotic therapy based on patient risk factors and clinical characteristics remains the mainstay of treatment for SAP (62). Research into potential prophylactics for SAP includes the use of Angiotensin-converting enzyme inhibitors, propranolol, caspase inhibitors and Cilostazol but is beyond the scope of this discussion (63, 64). For these patients, the rate of tracheostomy placement is high even with surgical decompression, near 30–40% (65, 66). Within the general critical care population there have been some benefits from early tracheostomy including fewer ventilation days and lower intensive care unit (ICU) length of stays (67). In the neuro critical care population, which tracheostomy is needed for orophayngeal weakness, the SETPOINT trial demonstrated reduction in sedative use, ICU mortality, and 6-month mortality (68). In a single center retrospective review of malignant hemispheric stroke patients that undergo surgical decompression, early tracheostomy was associated with reduction in mortality, ICU length of stay, mechanical ventilation days and pneumonia (65). The follow-up study, SETPOINT2 was a multi-center study of 366 patients randomized to early (4 days) vs. late (11 days) after intubation and did not find a statistically significant difference in severe disability (mRS 0–4) at 6 months (69). The authors did note that the wide confidence intervals around the effect estimate may encompass a clinically important difference making it difficult to exclude a clinically relevant benefit or harm from early tracheostomy.

Early initiation of antiplatelets and chemical venous thromboembolism prophylaxis is standard of care if not otherwise contraindicated (31). For patients with LHI who are potential surgical candidates, it is reasonable to defer initiation of full dose anticoagulation until surgical intervention has been excluded. Often this includes discontinuation of dual antiplatelet therapies, but it is generally recommended and safe to continue aspirin alone (33). Prophylactic doses of heparin or low molecular weight heparin are important to continue even in the setting of possible decline requiring surgical intervention as the risk of DVT is high in the stroke population (33, 70–72) and can be reversed prior surgical interventions.

## Communication and shared decision-making

It is important to initiate early discussions with patients and or healthcare decision makers regarding the expected progression of cerebral edema and critical care course. As discussed elsewhere in this issue, the beneficial window for surgical intervention is small. The greatest potential for surgical benefit is within 48 h of stroke onset (10, 73). Surrogates report the time pressure associated with acute stroke decision-making can lead to treatment or deferral of treatment that may be inconsistent with patient values (74). Decision-makers can benefit from early discussions to fully clarify the risks, benefits, and set expectations for neurological recovery prior to pressured decision-making, especially regarding surgery. Delayed surgical intervention for hemispheric stroke, defined as after 4 days, demonstrates benefits in survival but more patients have significant disability (5, 75). For patients who are initially medically managed, it is important to communicate that surgical interventions such as delayed hemicraniectomy, tracheostomy and gastrostomy tube placement are life prolonging measures and do not reverse functional disability. Clinicians should be aware of the limitations of prognostic scales which are based on population data and may not reflect the important outcomes or values to the specific patient and their surrogate decision-makers (76). Caution should also be taken by the clinician when discussing dichotomized outcomes such as favorable vs. unfavorable, as studies demonstrate discordance between clinician, patient and provider perceptions of acceptable quality of life (77, 78). Early limitations, such as new do-not-resuscitate orders changed within the first 24 h of acute brain injury led to less aggressive care and higher mortality (79–82). Therefore, early discussions in LHI are important in developing a shared understanding of the values of the patient and ensuring surrogate decision-makers are able to ask questions, reflect on treatment options, and pursue decisions most aligned with patient and family values.

## Conclusion

Cerebral edema following large hemispheric infarct can progress to life threatening herniation requiring medical and surgical interventions. Some patients may benefit from early surgical decompression, but others may not be considered candidates due to age, delayed presentation, or medical co-morbidities. For these patients, osmotherapy is frequently employed for neurologic decline presumed to be due to elevated ICP or cerebral edema. Robust data demonstrating hyperosmolar therapy changes functional outcomes is lacking. Glibenclamide may be a promising therapeutic option to mitigate the progression of cerebral edema in LHI. Important attention to critical care management can prevent or reduce morbidity from stroke related complications.

## Author contributions

GD was involved in the literature search, writing, and editing of the manuscript. WL was involved in the conceptualization, writing, and editing of the manuscript. Both authors contributed to the article and approved the submitted version.

## Conflict of interest

Author WL is a site sub-investigator in 2 Biogen studies of Glibenclamide in acute brain injury, but not the principal Investigator and does not receive direct funding from the study sponsor. The remaining author declares that the research was conducted in the absence of any commercial or financial relationships that could be construed as a potential conflict of interest

## Publisher's note

All claims expressed in this article are solely those of the authors and do not necessarily represent those of their affiliated organizations, or those of the publisher, the editors and the reviewers. Any product that may be evaluated in this article, or claim that may be made by its manufacturer, is not guaranteed or endorsed by the publisher.

## References

[1] Virani SS Alonso A Benjamin EJ Bittencourt MS Callaway CW Carson AP. Heart disease and stroke statistics-2020 update: a report from the American heart association. Circulation. (2020) 141:e139–596. 10.1161/CIR.0000000000000757 31992061

[2] Hacke W Schwab S Horn M Spranger M De Georgia M von Kummer R. “Malignant” middle cerebral artery territory infarction: clinical course and prognostic signs. Arch Neurol. (1996) 53:309–15. 10.1001/archneur.1996.00550040037012 8929152

[3] Vahedi K Vicaut E Mateo J Kurtz A Orabi M Guichard J-P. Sequential-design, multicenter, randomized, controlled trial of early decompressive craniectomy in malignant middle cerebral artery infarction (DECIMAL Trial). Stroke. (2007) 38:2506–17. 10.1161/STROKEAHA.107.485235 17690311

[4] Jüttler E Schwab S Schmiedek P Unterberg A Hennerici M Woitzik J. Decompressive surgery for the treatment of malignant infarction of the middle cerebral artery (DESTINY): a randomized, controlled trial. Stroke. (2007) 38:2518–25. 10.1161/STROKEAHA.107.485649 17690310

[5] Hofmeijer J Kappelle LJ Algra A Amelink GJ van Gijn J van der Worp HB. Surgical decompression for space-occupying cerebral infarction (the hemicraniectomy after middle cerebral artery infarction with life-threatening edema trial [HAMLET]): a multicentre, open, randomised trial. Lancet Neurol. (2009) 8:326–33. 10.1016/S1474-4422(09)70047-X 19269254

[6] Heinsius T Bogousslavsky J Van Melle G. Large infarcts in the middle cerebral artery territory. Etiology and outcome patterns. Neurology. (1998) 50:341–50. 10.1212/WNL.50.2.341 9484351

[7] Huang X Yang Q Shi X Xu X Ge L Ding X. Predictors of malignant brain edema after mechanical thrombectomy for acute ischemic stroke. J Neurointerv Surg. (2019) 11:994–8. 10.1136/neurintsurg-2018-014650 30798266

[8] Wu S Yuan R Wang Y Wei C Zhang S Yang X. Early prediction of malignant brain edema after ischemic stroke. Stroke. (2018) 49:2918–27. 10.1161/STROKEAHA.118.022001 30571414

[9] Qureshi AI Suarez JI Yahia AM Mohammad Y Uzun G Suri MFK. Timing of neurologic deterioration in massive middle cerebral artery infarction: a multicenter review. Crit Care Med. (2003) 31:272–7. 10.1097/00003246-200301000-00043 12545028

[10] Reinink H Jüttler E Hacke W Hofmeijer J Vicaut E Vahedi K. Surgical decompression for space-occupying hemispheric infarction: a systematic review and individual patient meta-analysis of randomized clinical trials. JAMA Neurol. (2021) 78:208–16. 10.1001/jamaneurol.2020.3745 33044488 PMC7551237

[11] Kelly AG Zahuranec DB Holloway RG Morgenstern LB Burke JF. Variation in do-not-resuscitate orders for patients with ischemic stroke: implications for national hospital comparisons. Stroke. (2014) 45:822–7. 10.1161/STROKEAHA.113.004573 24523035 PMC3943476

[12] Simard JM Kent TA Chen M Tarasov KV Gerzanich V. Brain oedema in focal ischaemia: molecular pathophysiology and theoretical implications. Lancet Neurol. (2007) 6:258–68. 10.1016/S1474-4422(07)70055-8 17303532 PMC2725365

[13] Pergakis M Badjatia N Chaturvedi S Cronin CA Kimberly WT Sheth KN. BIIB093 (IV glibenclamide): an investigational compound for the prevention and treatment of severe cerebral edema. Expert Opin Investig Drugs. (2019) 28:1031–40. 10.1080/13543784.2019.1681967 31623469 PMC6893098

[14] Simard JM Chen M Tarasov KV Bhatta S Ivanova S Melnitchenko L. Newly expressed SUR1-regulated NC_Ca − ATP_ channel mediates cerebral edema after ischemic stroke. Nat Med. (2006) 12:433–40. 10.1038/nm1390 16550187 PMC2740734

[15] Barros LF Hermosilla T Castro J. Necrotic volume increase and the early physiology of necrosis. Comp Biochem Physiol A Mol Integr Physiol. (2001) 130:401–9. 10.1016/S1095-6433(01)00438-X 11913453

[16] Solenov E Watanabe H Manley GT Verkman AS. Sevenfold-reduced osmotic water permeability in primary astrocyte cultures from AQP-4-deficient mice, measured by a fluorescence quenching method. Am J Physiol Cell Physiol. (2004) 286:C426–32. 10.1152/ajpcell.00298.2003 14576087

[17] Roberts WG Palade GE. Increased microvascular permeability and endothelial fenestration induced by vascular endothelial growth factor. J Cell Sci. (1995) 108:2369–79. 10.1242/jcs.108.6.2369 7673356

[18] Unemori EN Bouhana KS Werb Z. Vectorial secretion of extracellular matrix proteins, matrix-degrading proteinases, and tissue inhibitor of metalloproteinases by endothelial cells. J Biol Chem. (1990) 265:445–51. 10.1016/S0021-9258(19)40250-0 2152926

[19] Pfefferkorn T Rosenberg GA. Closure of the blood-brain barrier by matrix metalloproteinase inhibition reduces rtPA-mediated mortality in cerebral ischemia with delayed reperfusion. Stroke. (2003) 34:2025–30. 10.1161/01.STR.0000083051.93319.28 12855824

[20] Cook AM Morgan Jones G Hawryluk GWJ Mailloux P McLaughlin D Papangelou A. Guidelines for the acute treatment of cerebral edema in neurocritical care patients. Neurocrit Care. (2020) 32:647–66. 10.1007/s12028-020-00959-7 32227294 PMC7272487

[21] Mestre H Du T Sweeney AM Liu G Samson AJ Peng W. Cerebrospinal fluid influx drives acute ischemic tissue swelling. Science. (2020) 367:eaax7171. 10.1126/science.aax7171 32001524 PMC7375109

[22] Shimoyama T Kimura K Uemura J Yamashita S Saji N Shibazaki K. The DASH score: a simple score to assess risk for development of malignant middle cerebral artery infarction. J Neurol Sci. (2014) 338:102–6. 10.1016/j.jns.2013.12.024 24423583

[23] Ong CJ Gluckstein J Laurido-Soto O Yan Y Dhar R Lee J-M. Enhanced detection of edema in malignant anterior circulation stroke (EDEMA) score: a risk prediction tool. Stroke. (2017) 48:1969–72. 10.1161/STROKEAHA.117.016733 28487333 PMC5487281

[24] Muscari A Faccioli L Lega MV Lorusso A Trossello MP Puddu GM. Predicting cerebral edema in ischemic stroke patients. Neurol Sci. (2019) 40:745–52. 10.1007/s10072-019-3717-y 30659418

[25] Cheng Y Wu S Wang Y Song Q Yuan R Wu Q. External validation and modification of the EDEMA score for predicting malignant brain edema after acute ischemic stroke. Neurocrit Care. (2020) 32:104–12. 10.1007/s12028-019-00844-y 31549349

[26] Kimberly WT Dutra BG Boers AMM Alves HCBR Berkhemer OA van den Berg L. Association of reperfusion with brain edema in patients with acute ischemic stroke: a secondary analysis of the MR CLEAN trial. JAMA Neurol. (2018) 75:453–61. 10.1001/jamaneurol.2017.5162 29365017 PMC5885187

[27] Broocks G Flottmann F Scheibel A Aigner A Faizy TD Hanning U. Quantitative lesion water uptake in acute stroke computed tomography is a predictor of malignant infarction. Stroke. (2018) 49:1906–12. 10.1161/STROKEAHA.118.020507 29976584

[28] Dhar R Yuan K Kulik T Chen Y Heitsch L An H. CSF volumetric analysis for quantification of cerebral edema after hemispheric infarction. Neurocrit Care. (2016) 24:420–7. 10.1007/s12028-015-0204-z 26438467 PMC4821820

[29] Dhar R Chen Y Hamzehloo A Kumar A Heitsch L He J. Reduction in cerebrospinal fluid volume as an early quantitative biomarker of cerebral edema after ischemic stroke. Stroke. (2020) 51:462–7. 10.1161/STROKEAHA.119.027895 31818229 PMC6991889

[30] Foroushani HM Hamzehloo A Kumar A Chen Y Heitsch L Slowik A. Quantitative serial CT imaging-derived features improve prediction of malignant cerebral edema after ischemic stroke. Neurocrit Care. (2020) 33:785–92. 10.1007/s12028-020-01056-5 32729090 PMC7738418

[31] Powers WJ Rabinstein AA Ackerson T Adeoye OM Bambakidis NC Becker K. Guidelines for the early management of patients with acute ischemic stroke: 2019 update to the 2018 guidelines for the early management of acute ischemic stroke: a guideline for healthcare professionals from the American heart association/American stroke association. Stroke. (2019) 50:e344–418. 10.1161/STR.0000000000000211 31662037

[32] Bektas H Wu T-C Kasam M Harun N Sitton CW Grotta JC. Increased blood-brain barrier permeability on perfusion CT might predict malignant middle cerebral artery infarction. Stroke. (2010) 41:2539–44. 10.1161/STROKEAHA.110.591362 20847316 PMC3412880

[33] Wijdicks EFM Sheth KN Carter BS Greer DM Kasner SE Kimberly WT. Recommendations for the management of cerebral and cerebellar infarction with swelling: a statement for healthcare professionals from the American heart association/American stroke association. Stroke. (2014) 45:1222–38. 10.1161/01.str.0000441965.15164.d6 24481970

[34] van der Worp HB Hofmeijer J Jüttler E Lal A Michel P Santalucia P. European stroke organisation (ESO) guidelines on the management of space-occupying brain infarction. Eur Stroke J. (2021). 10.1177/23969873211014112 34414304 PMC8370077

[35] Cardim D Robba C Bohdanowicz M Donnelly J Cabella B Liu X. Non-invasive monitoring of intracranial pressure using transcranial doppler ultrasonography: is it possible? Neurocrit Care. (2016) 25:473–91. 10.1007/s12028-016-0258-6 26940914 PMC5138275

[36] Alsallom F Casassa C Akkineni K Lin L. Early detection of cerebral herniation by continuous electroencephalography and quantitative analysis. Clin EEG Neurosci. (2022) 53:133–7. 10.1177/15500594211018535 34028297

[37] Videen TO Zazulia AR Manno EM Derdeyn CP Adams RE Diringer MN. Mannitol bolus preferentially shrinks non-infarcted brain in patients with ischemic stroke. Neurology. (2001) 57:2120–2. 10.1212/WNL.57.11.2120 11739839

[38] Surani S Lockwood G Macias MY Guntupalli B Varon J. Hypertonic saline in elevated intracranial pressure: past, present, and future. J Intensive Care Med. (2015) 30:8–12. 10.1177/0885066613487151 23753247

[39] Muizelaar JP Lutz HA Becker DP. Effect of mannitol on ICP and CBF and correlation with pressure autoregulation in severely head-injured patients. J Neurosurg. (1984) 61:700–6. 10.3171/jns.1984.61.4.0700 6432972

[40] Muizelaar JP Wei EP Kontos HA Becker DP. Mannitol causes compensatory cerebral vasoconstriction and vasodilation in response to blood viscosity changes. J Neurosurg. (1983) 59:822–8. 10.3171/jns.1983.59.5.0822 6413661

[41] Diringer MN Scalfani MT Zazulia AR Videen TO Dhar R Powers WJ. Effect of mannitol on cerebral blood volume in patients with head injury. Neurosurgery. (2012) 70:1215–8; discussion 1219. 10.1227/NEU.0b013e3182417bc2 22089753 PMC3727970

[42] García-Morales EJ Cariappa R Parvin CA Scott MG Diringer MN. Osmole gap in neurologic-neurosurgical intensive care unit: Its normal value, calculation, and relationship with mannitol serum concentrations. Crit Care Med. (2004) 32:986–91. 10.1097/01.CCM.0000120057.04528.60 15071390

[43] Chen H Song Z Dennis JA. Hypertonic saline versus other intracranial pressure-lowering agents for people with acute traumatic brain injury. Cochrane Database Syst Rev. (2020) 1:CD010904. 10.1002/14651858.CD010904.pub3 31978260 PMC6984412

[44] Schwimmbeck F Voellger B Chappell D Eberhart L. Hypertonic saline versus mannitol for traumatic brain injury: a systematic review and meta-analysis with trial sequential analysis. J Neurosurg Anesthesiol. (2021) 33:10–20. 10.1097/ANA.0000000000000644 31567726

[45] Strandvik GF. Hypertonic saline in critical care: a review of the literature and guidelines for use in hypotensive states and raised intracranial pressure. Anaesthesia. (2009) 64:990–1003. 10.1111/j.1365-2044.2009.05986.x 19686485

[46] Lawson T Hussein O Nasir M Hinduja A Torbey MT. Intraosseous administration of hypertonic saline in acute brain-injured patients: a prospective case series and literature review. Neurologist. (2019) 24:176–9. 10.1097/NRL.0000000000000248 31688708 PMC6839785

[47] Wang J Fang Y Ramesh S Zakaria A Putman MT Dinescu D. Intraosseous administration of 234% Nacl for treatment of intracranial hypertension. Neurocrit Care. (2019) 30:364–71. 10.1007/s12028-018-0637-2 30397844

[48] Mason A Malik A Ginglen JG. Hypertonic Fluids. StatPearls Treasure Island, FL: StatPearls Publishing (2022). 31194351

[49] Roquilly A Moyer JD Huet O Lasocki S Cohen B Dahyot-Fizelier C. Effect of continuous infusion of hypertonic saline vs standard care on 6-month neurological outcomes in patients with traumatic brain injury: the COBI randomized clinical trial. JAMA. (2021) 325:2056–66. 10.1001/jama.2021.5561 34032829 PMC8150692

[50] Simard JM Woo SK Schwartzbauer GT Gerzanich V. Sulfonylurea receptor 1 in central nervous system injury: a focused review. J Cereb Blood Flow Metab. (2012) 32:1699–717. 10.1038/jcbfm.2012.91 22714048 PMC3434627

[51] Stokum JA Kwon MS Woo SK Tsymbalyuk O Vennekens R Gerzanich V. SUR1-TRPM4 and AQP4 form a heteromultimeric complex that amplifies ion/water osmotic coupling and drives astrocyte swelling. Glia. (2018) 66:108–25. 10.1002/glia.23231 28906027 PMC5759053

[52] Sheth KN Elm JJ Molyneaux BJ Hinson H Beslow LA Sze GK. Safety and efficacy of intravenous glyburide on brain swelling after large hemispheric infarction (GAMES-RP): a randomised, double-blind, placebo-controlled phase 2 trial. Lancet Neurol. (2016) 15:1160–9. 10.1016/S1474-4422(16)30196-X 27567243

[53] Kimberly WT Bevers MB von Kummer R Demchuk AM Romero JM Elm JJ. Effect of IV glyburide on adjudicated edema endpoints in the GAMES-RP trial. Neurology. (2018) 91:e2163–9. 10.1212/WNL.0000000000006618 30446594 PMC6282228

[54] Vorasayan P Bevers MB Beslow LA Sze G Molyneaux BJ Hinson HE. Intravenous glibenclamide reduces lesional water uptake in large hemispheric infarction. Stroke. (2019) 50:3021–7. 10.1161/STROKEAHA.119.026036 31537189 PMC6817419

[55] Hinson HE Sun E Molyneaux BJ von Kummer R Demchuk A Romero J. Osmotherapy for malignant cerebral edema in a phase 2 prospective, double blind, randomized, placebo-controlled study of IV glibenclamide. J Stroke Cerebrovasc Dis. (2020) 29:104916. 10.1016/j.jstrokecerebrovasdis.2020.104916 32414580

[56] Vakili A Kataoka H Plesnila N. Role of arginine vasopressin V1 and V2 receptors for brain damage after transient focal cerebral ischemia. J Cereb Blood Flow Metab. (2005) 25:1012–9. 10.1038/sj.jcbfm.9600097 15744246

[57] Stokum JA Gerzanich V Sheth KN Kimberly WT Simard JM. Emerging pharmacological treatments for cerebral edema: evidence from clinical studies. Annu Rev Pharmacol Toxicol. (2020) 60:291–309. 10.1146/annurev-pharmtox-010919-023429 31914899 PMC7122796

[58] Stocchetti N Maas AIR Chieregato A van der Plas AA. Hyperventilation in head injury: a review. Chest. (2005) 127:1812–27. 10.1378/chest.127.5.1812 15888864

[59] Mavrocordatos P Bissonnette B Ravussin P. Effects of neck position and head elevation on intracranial pressure in anaesthetized neurosurgical patients: preliminary results. J Neurosurg Anesthesiol. (2000) 12:10–4. 10.1097/00008506-200001000-00003 10636614

[60] Sandercock PA Soane T. Corticosteroids for acute ischaemic stroke. Cochrane Database Syst Rev. (2011) 2011:CD000064. 10.1002/14651858.CD000064.pub2 21901674 PMC7032681

[61] Teasell RW McRae M Marchuk Y Finestone HM. Pneumonia associated with aspiration following stroke. Arch Phys Med Rehabil. (1996) 77:707–9. 10.1016/S0003-9993(96)90012-X 8669999

[62] Hannawi Y Hannawi B Rao CPV Suarez JI Bershad EM. Stroke-associated pneumonia: major advances and obstacles. Cerebrovasc Dis. (2013) 35:430–43. 10.1159/000350199 23735757

[63] Prass K Meisel C Höflich C Braun J Halle E Wolf T. Stroke-induced immunodeficiency promotes spontaneous bacterial infections and is mediated by sympathetic activation reversal by poststroke T helper cell type 1-like immunostimulation. J Exp Med. (2003) 198:725–36. 10.1084/jem.20021098 12939340 PMC2194193

[64] Prass K Braun JS Dirnagl U Meisel C Meisel A. Stroke propagates bacterial aspiration to pneumonia in a model of cerebral ischemia. Stroke. (2006) 37:2607–12. 10.1161/01.STR.0000240409.68739.2b 16946159

[65] Qureshi MSS Shad ZS Shoaib F Munawar K Saeed ML Hussain SW. Early versus late tracheostomy after decompressive craniectomy. Cureus. (2018) 10:e3699. 10.7759/cureus.3699 30788188 PMC6372250

[66] Walcott BP Kamel H Castro B Kimberly WT Sheth KN. Tracheostomy after severe ischemic stroke: a population-based study. J Stroke Cerebrovasc Dis. (2014) 23:1024–9. 10.1016/j.jstrokecerebrovasdis.2013.08.019 24103666 PMC3976897

[67] Wahlster S Sharma M Chu F Granstein JH Johnson NJ Longstreth WT. Outcomes after tracheostomy in patients with severe acute brain injury: a systematic review and meta-analysis. Neurocrit Care. (2021) 34:956–67. 10.1007/s12028-020-01109-9 33033959 PMC8363498

[68] Bösel J Schiller P Hook Y Andes M Neumann J-O Poli S. Stroke-related early tracheostomy versus prolonged orotracheal intubation in neurocritical care trial (SETPOINT): a randomized pilot trial. Stroke. (2013) 44:21–8. 10.1161/STROKEAHA.112.669895 23204058

[69] Bösel J Niesen W-D Salih F Morris NA Ragland JT Gough B. Effect of early vs standard approach to tracheostomy on functional outcome at 6 months among patients with severe stroke receiving mechanical ventilation: the SETPOINT2 randomized clinical trial. JAMA. (2022) 327:1899–909. 10.1001/jama.2022.4798 35506515 PMC9069344

[70] Douds GL Hellkamp AS Olson DM Fonarow GC Smith EE Schwamm LH. Venous thromboembolism in the get with the guidelines-stroke acute ischemic stroke population: incidence and patterns of prophylaxis. J Stroke Cerebrovasc Dis. (2014) 23:123–9. 10.1016/j.jstrokecerebrovasdis.2012.10.018 23253528

[71] Warlow C Ogston D Douglas AS. Deep venous thrombosis of the legs after strokes. Part I–incidence and predisposing factors. Br Med J. (1976) 1:1178–81. 10.1136/bmj.1.6019.1178 1268614 PMC1639761

[72] Warlow C Ogston D Douglas AS. Venous thrombosis following strokes. Lancet. (1972) 1:1305–6. 10.1016/S0140-6736(72)91034-3 4113399

[73] Vahedi K Hofmeijer J Juettler E Vicaut E George B Algra A. Early decompressive surgery in malignant infarction of the middle cerebral artery: a pooled analysis of three randomised controlled trials. Lancet Neurol. (2007) 6:215–22. 10.1016/S1474-4422(07)70036-4 17303527

[74] de Boer ME Depla M Wojtkowiak J Visser MC Widdershoven GAM Francke AL. Life-and-death decision-making in the acute phase after a severe stroke: interviews with relatives. Palliat Med. (2015) 29:451–7. 10.1177/0269216314563427 25634632

[75] Geurts M van der Worp HB Kappelle LJ Amelink GJ Algra A Hofmeijer J. Surgical decompression for space-occupying cerebral infarction: outcomes at 3 years in the randomized HAMLET trial. Stroke. (2013) 44:2506–8. 10.1161/STROKEAHA.113.002014 23868265

[76] Gao L Zhao CW Hwang DY. End-of-Life Care Decision-Making in Stroke. Front Neurol. (2021) 12:702833. 10.3389/fneur.2021.702833 34650502 PMC8505717

[77] Neugebauer H Creutzfeldt CJ Hemphill JC Heuschmann PU Jüttler E. DESTINY-S: attitudes of physicians toward disability and treatment in malignant MCA infarction. Neurocrit Care. (2014) 21:27–34. 10.1007/s12028-014-9956-0 24549936

[78] Kiphuth IC Köhrmann M Lichy C Schwab S Huttner HB. Hemicraniectomy for malignant middle cerebral artery infarction: retrospective consent to decompressive surgery depends on functional long-term outcome. Neurocrit Care. (2010) 13:380–4. 10.1007/s12028-010-9449-8 20890678

[79] Madhok DY Vitt JR MacIsaac D Hsia RY Kim AS Hemphill JC. Early do-not-resuscitate orders and outcome after intracerebral hemorrhage. Neurocrit Care. (2021) 34:492–9. 10.1007/s12028-020-01014-1 32661793

[80] Minhas JS Sammut-Powell C Birleson E Patel HC Parry-Jones AR. Are do-not-resuscitate orders associated with limitations of care beyond their intended purpose in patients with acute intracerebral haemorrhage? analysis of the ABC-ICH study. BMJ Open Qual. (2021) 10:e001113. 10.1136/bmjoq-2020-001113 33547153 PMC7871257

[81] Reinink H Konya B Geurts M Kappelle LJ van der Worp HB. Treatment restrictions and the risk of death in patients with ischemic stroke or intracerebral hemorrhage. Stroke. (2020) 51:2683–9. 10.1161/STROKEAHA.120.029788 32757755

[82] Hemphill JC Newman J Zhao S Johnston SC. Hospital usage of early do-not-resuscitate orders and outcome after intracerebral hemorrhage. Stroke. (2004) 35:1130–4. 10.1161/01.STR.0000125858.71051.ca 15044768

